# Prospects of the Use of Cell Therapy to Induce Immune Tolerance

**DOI:** 10.3389/fimmu.2020.00792

**Published:** 2020-05-12

**Authors:** Zhenkun Wang, Xiaolong Liu, Fenglin Cao, Joseph A. Bellanti, Jin Zhou, Song Guo Zheng

**Affiliations:** ^1^Central Laboratory of Hematology and Oncology, First Affiliated Hospital, Harbin Medical University, Harbin, China; ^2^College of Life Science, Northeast Agricultural University, Harbin, China; ^3^Departments of Pediatrics and Microbiology-Immunology, The International Center for Interdisciplinary Studies of Immunology (ICISI), Georgetown University Medical Center, Washington, DC, United States; ^4^Department of Hematology, First Affiliated Hospital, Harbin Medical University, Harbin, China; ^5^Department of Internal Medicine, Ohio State University College of Medicine, Columbus, OH, United States

**Keywords:** autoimmune diseases, immune homeostasis, cell therapy, immune tolerance, immune reconstitution

## Abstract

Conditions in which abnormal or excessive immune responses exist, such as autoimmune diseases (ADs), graft-versus-host disease, transplant rejection, and hypersensitivity reactions, are serious hazards to human health and well-being. The traditional immunosuppressive drugs used to treat these conditions can lead to decreased immune function, a higher risk of infection, and increased tumor susceptibility. As an alternative therapeutic approach, cell therapy, in which generally intact and living cells are injected, grafted, or implanted into a patient, has the potential to overcome the limitations of traditional drug treatment and to alleviate the symptoms of many refractory diseases. Cell therapy could be a powerful approach to induce immune tolerance and restore immune homeostasis with a deeper understanding of immune tolerance mechanisms and the development of new techniques. The purpose of this review is to describe the current panoramic scope of cell therapy for immune-mediated disorders, discuss the advantages and disadvantages of different types of cell therapy, and explore novel directions and future prospects for these tolerogenic therapies.

## Immune Homeostasis

**Figure 1:**
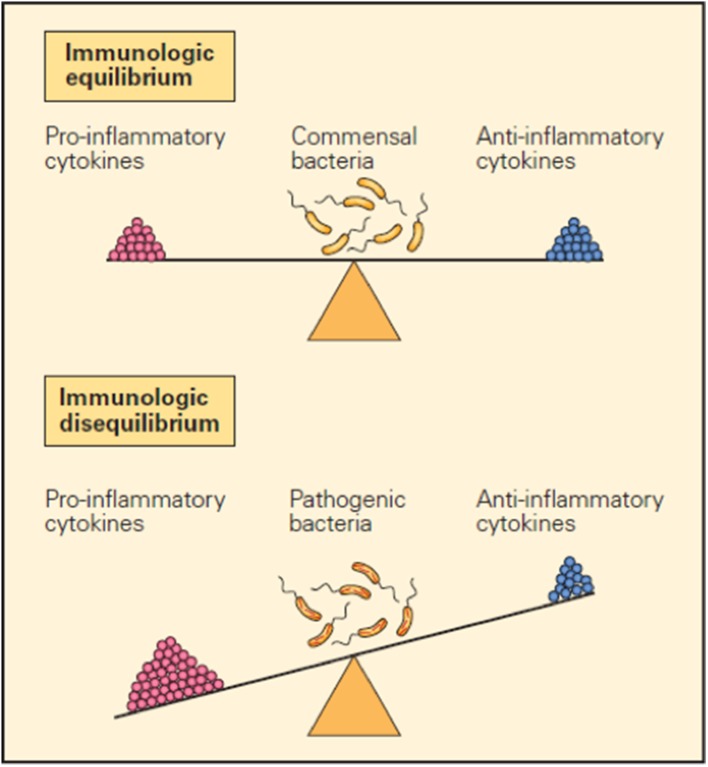
Schematic representation of the opposing pro- and anti-inflammatory functions of cytokines in maintaining immunological equilibrium. A balanced production of pro- and anti-inflammatory cytokines in response to a foreign configuration is one of the mechanisms that contribute to immunological equilibrium (upper panel). Conversely, immunological imbalance or disease is seen when there is overproduction of pro-inflammatory cytokines and/or inadequate production of anti-inflammatory cytokines (lower panel) (1).

It is now clear that many factors such as cytokines, immune cells, immune checkpoints, and microbiota are important in maintaining the immune balance between the external and internal environment and that they accomplish their function by either promoting or inhibiting inflammatory responses (1–3). For example, balanced production of pro- and anti-inflammatory cytokines in response to a foreign configuration results in immunological equilibrium and defines the immune system in health in contrast to the immunologic imbalance seen when an overproduction of pro-inflammatory cytokines and/or inadequate production of anti-inflammatory cytokines creates disequilibrium, which represents the immune system in disease (Figure 1).

**Figure 2:**
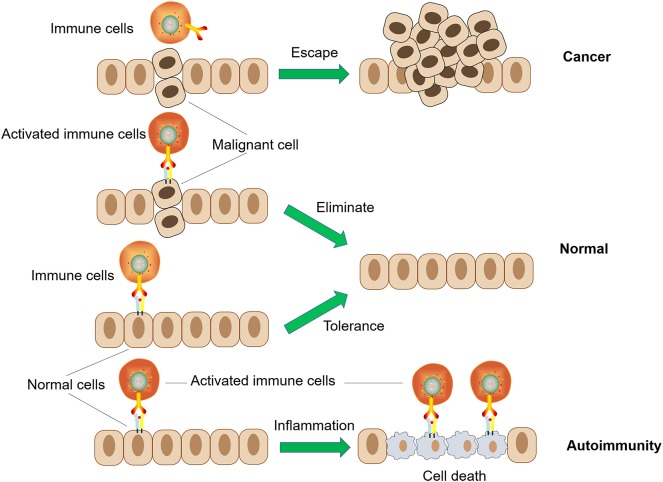
Two types of immune dysfunction: cancer and autoimmunity.

A healthy immune system should, therefore, exist in a state of dynamic equilibrium, with a balance between pro-inflammatory and anti-inflammatory responses, to achieve immune homeostasis. Ideally, the immune system normally maintains its non-responsiveness to autoantigens and various harmless environmental entities (allergens, commensal microbiota, etc.) through complex mechanisms of central and peripheral tolerance. During the course of pathogen invasion or the appearance of a malignant proliferation of autologous cells, the body needs to recognize and generate a targeted and effective immune response to the abnormality. After successfully eliminating these deleterious challenges, the immune response normally self-adjusts and returns to its beneficial state of immune homeostasis. However, if immune homeostasis is not effectively achieved, continuous immune hyperactivity or inhibition might contribute to two types of disease occurrence: autoimmune disease or cancer (4–7). On the other hand, the hypo-functioning immune system with deficiencies in immune recognition or effects will increase the possibility of infection, chronic inflammation, and tumors. Additionally, abnormal autoimmune responses to autoantigens might occur if the central tolerance or peripheral tolerance of the immune system is damaged (Figure 2) (8, 9). As two types of contrasting immune dysfunction disorders, their different mechanisms of pathogenesis can serve as a useful reference for designing counter-treatment strategies (10). For example, tumor cells are “altered cells” and are generally perceived as “self” and non-dangerous, with the privileges of normal healthy tissues. Treating autoimmune diseases might help to understand how tumor cells lose these properties, making them target the immune system, and gain such properties, making them appear non-dangerous (11).

In addition to autoimmune diseases (ADs), excessive inflammatory reactions caused by organ transplantation and hypersensitivity reactions can be life-threatening for patients. Therefore, restoring immune tolerance to autoantigens or inducing immune tolerance to specific antigens is of considerable importance for the clinical treatment of many diseases (12, 13).

Autoimmune responses are associated with imbalances between effector and inhibitory cells. When a pathological immune response begins, the effector cells in the tissue accumulate, eliciting an autoimmune response, which may be accompanied by a relative decrease in the number of inhibitory cells or an increased number of dysfunctional inhibitory cells (14). Imbalances in effector and regulatory cells can lead to autoimmunity or even tissue damage, and the patients often experience remission and recurrence due to persistent interactions between pathogenic responses and regulation (15). Thus, the development of strategies to eliminate autoimmune responses and recover the balance of effector and regulatory cells is becoming an important task for achieving immune tolerance and disease prevention.

Traditional drugs are often limited in their ability to induce immune tolerance due to complex immunoregulatory processes (16). Since cell–cell interactions are fundamental for immune responses, cell-based therapeutic techniques have developed rapidly (17, 18) as alternative approaches to traditional drugs. Cell therapy has become the “hot topic” in cancer research; in particular, chimeric antigen receptor T (CAR-T) cells have made significant breakthroughs in the treatment of hematologic malignancies (19, 20). In contrast, cell therapy for ADs has progressed slowly in recent years. Although cytokine antagonists have altered the course of many ADs, most current therapeutic agents target the terminal phase of inflammation and do not address the fundamental problems that are responsible for the initiation and progression of the autoimmune process (15). ADs are often characterized by chronic conditions, which in most cases require sustained, even life-long treatment, which imposes a heavy strain and financial burden on patients and significantly increases their risk of malignant tumors and infectious complications (21–23). As cell immune therapy technology can induce immune tolerance through various immunosuppressive pathways and is expected to achieve long-term relief of the disease (24–26), it is becoming a promising technology in the field of immune tolerability.

## Cell Therapy Approaches to ADs

ADs comprise more than 80 different immune disorders including rheumatoid arthritis (RA), type 1 diabetes (T1DM), systemic lupus erythematosus (SLE), multiple sclerosis (MS), Amyotrophic lateral sclerosis (ALS), etc., which collectively affect ~4.5% of the population (27, 28). The consensus is that a mixture of genetic susceptibility and environmental factors are involved in the development of autoimmunity. Large-scale genome-wide association studies (GWASs) and their meta-analyses with a large sample size have elucidated the disease-susceptible genes and disease-causing pathways of many ADs (29). However, potential target autoantigens remain unknown in some of the most important human ADs, and the precise mechanism of disease pathogenesis remains elusive (30).

ADs are generally characterized by the presence of autoreactive immune lymphocytes, leading to intermittent or sustained inflammation (15). In terms of genetic factors, typically, polymorphic HLA genes are associated with antigen presentation pathways, and gene polymorphisms of *FOXP3*, immune checkpoints, cytokines, and cytokine receptors are all related to immune regulation (31–34). In terms of environmental factors, smoking and intestinal microbes, among others, have been implicated in triggering autoimmunity reactions (28, 35–39). A simple hypothesis that has been put forward to explain the pathogenesis of autoimmune disease is that polymorphisms in various genes result in defective regulation or reduced thresholds for lymphocyte activation, and environmental factors initiate or augment the activation of self-reactive lymphocytes that have escaped control and are poised to react against self-constituents (15). A combination of triggering factors leads to the diversity of immune disorders, including cellular immunity, humoral immunity, neuroendocrine networks, and cytokine networks (21). There are individual differences in immune disorder indicators among different patients, and the immune disorder status of the same patient can change as the AD progresses, presenting a difficult clinical challenge for the treatment of complex, individualized conditions. These different modes of pathogenesis combined with different courses of disease progression are often associated with treatment failure, particularly when the patients are treated with a single drug regimen (40).

The occurrence and development of different ADs share the same common features of lost immune tolerance to autoantigens. Therefore, the fundamental way to treat the underlying problem of AD is to restore the patient's immune tolerance to autoantigens. The mechanisms through which cells maintain immune tolerance are diverse, and with the study of cellular functions and mechanisms, cell-based therapeutic techniques might provide broader prospects than conventional drugs. Several cell therapies have been developed and tested to treat ADs, allograft rejection, and graft-versus-host disease (GVHD).

## Strategy 1: Hematopoietic Stem Cell Transplantation (HSCT) for Immune Reconstitution

In 1977, Baldwin et al. (41) observed that RA symptoms were relieved in patients with gold salt-induced aplastic anemia (AA) following allogeneic bone marrow transplantation. In 1995, HSCT was clinically applied to severe ADs where conventional therapy had proved ineffective (42). There were two purposes for using HSCT: one was to eliminate autoreactive immune cells, and the other was to rebuild the self-tolerant immune system. Several clinical trials showed that autologous HSCT was superior to conventional treatments for the treatment of severe ADs, including multiple sclerosis and systemic sclerosis (43–46), and induces long-term disease remission without immunosuppressive drugs. However, some patients still experienced AD recurrence after transplantation (42, 47).

Most hematopoietic stem cells currently used to treat AD have been autologous using either an infusion of unpurified lymphocytes or immunopurified CD34^+^ HSCs. For several refractory ADs, autologous HSCT can be an effective treatment that induces long-term, drug-free, and asymptomatic remission (42). However, HSCT is still a high-risk treatment, with the occurrence of transplant failure, recurrence of disease, infection, and other complications that might decrease the survival rate of patients. Therefore, HSCT is only considered suitable for patients with serious disease and risk of death. It should be noted that incomplete eradication of autoreactive memory cells or the infusion of unpurified lymphocytes could cause disease recurrence. Encouragingly, a method for long-term *ex vivo* HSC expansion has been developed recently (48) that might make homogeneous HSCT easier. Moreover, some patients might develop disease flares resulting from the re-induction of autoimmunity driven by genetic predisposition, and allogeneic HSCT could be used as an alternative therapy (49). The further development of more effective and safer HSCT methods remains the next challenge in cell therapy so that this approach can be used more widely in the future for patients with ADs.

## Strategy 2: Adoptive Immunotherapy to Eliminate Autoreactive Immune Cells

**Figure 3:**
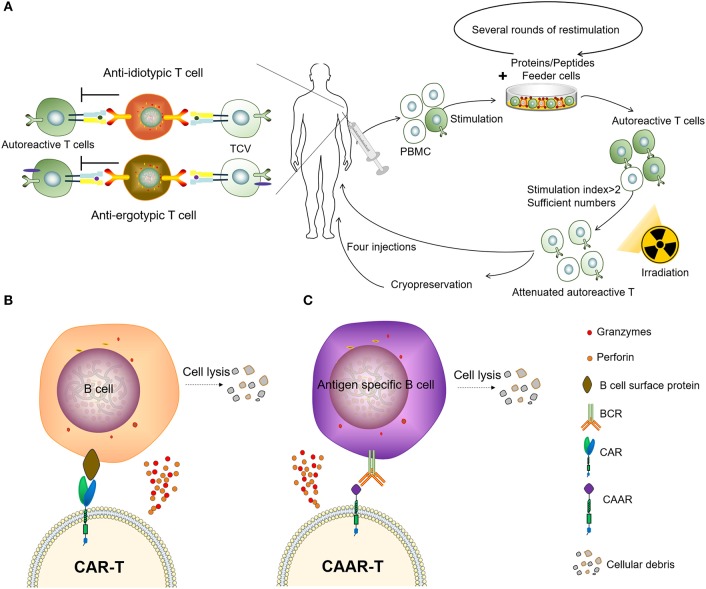
Two types of adoptive immunotherapy to eliminate autoreactive immune cells. **(A)** Patients receive TCV. **(B)** Chimeric antigen receptor T (CAR-T) cells targeting B-lineage antigens to kill all B cells. **(C)** Autoantigen-based chimeric immunoreceptors direct T cells to kill autoreactive B lymphocytes through the specificity of the B cell receptor (BCR).

Autoimmunity is characterized by the presence of autoantibodies and autoreactive T cells directed against normal components of an individual. T-cell vaccination (TCV) therapy is a type of autologous, personalized cell-based therapy in which attenuated autoreactive T cells are administered as immunogenic agents and targeted T-cells are deleted or inactivated (Figure 3A). The concept of TCV was first raised by Ben-nun et al. (50, 51) in 1981, based on the finding that irradiated T lymphocyte cells reactive against myelin basic protein (MBP) can induce a vaccination against experimental autoimmune encephalomyelitis (EAE). Vaccination with the attenuated anti-MBP T cells led to resistance to later attempts to induce EAE by active immunization to MBP in adjuvant (52). Subsequent research on the mechanisms of TCV has revealed a complicated anti-idiotypic and anti-ergotypic network to be responsible for the pathogenic procedure (53, 54). The subject responds to own vaccine T cells by activating regulatory networks of T cells, which, in turn, arrests the damaging inflammation that causes the autoimmune disease (55, 56). Over the past decades, the effect of TCV has been justified in several animal models of autoimmune diseases and graft rejection, including experimental autoimmune encephalomyelitis, lupus, autoimmune uveoretinitis, autoimmune diabetes, autoimmune thyroiditis, collagen-induced arthritis (CIA), and so on (57–62).

Moreover, TCV has shown safety and effectiveness in various clinical trials, mostly for patients with MS but also for RA, SLE, and ALS (63–66). Achiron et al. (67) evaluated the efficacy of TCV therapy for 20 patients with aggressive relapsing-remitting MS. TCV treatment had a favorable impact on both annual relapse rate and progression to disability. Seledtsova et al. (68) conducted a study where 39 patients with progressive (chronic) MS were multiply immunized with autological polyclonal TCVs. In the TCV-treated patients, sustained reduction in plasma IFN-γ levels and concomitant increases in IL-4 levels were documented. Indeed, polyclonal T-cell vaccination led to a considerable reduction of proliferative responses of T cells to myelin-associated antigens. Huang et al. (66) enrolled 16 patients with systemic lupus erythematosus (SLE). They found that TCV was associated with remissions in clinical symptoms, reductions in Systemic Lupus Erythematosus Disease Activity Index (SLEDAI) and anti-ds-DNA antibodies, and increases in complement component 3 (C3) and C4. In addition, it is helpful in lowering the glucocorticoid doses of patients' regular usage. Unfortunately, TCV has been somewhat ignored in the past due to standard pharmaceutical avoidance of cell-based and individualized treatments. Nonetheless, cell therapy appears to be coming of age, and TCV has been granted fast-track status by the FDA for the treatment of some types of multiple sclerosis (10).

The presence of autoantibodies is a feature of many ADs and has been widely used to aid the diagnosis of such diseases. B cell/plasma cells have been recognized as an important target for the treatment of some ADs (69). Several drugs that target B cells are in clinical use or are currently being developed, including monoclonal antibodies to target CD19, CD20, and CD22, which are expected to effectively treat various ADs (69). Rituximab depletes B cells by complement-dependent cytotoxicity (CDC) and antibody-dependent cellular cytotoxicity (ADCC) effects. This drug is now approved for the treatment of RA, granulomatosis with polyangiitis (GPA), and microscopic polyangiitis (MPA), as well as several systemic inflammatory autoimmune diseases (SIADs) (70, 71). However, such antibody therapy requires repeated administration, and drug resistance can occur during long-term administration (72).

The CAR-T has been a major breakthrough for the treatment of B cell malignancies, and these CAR-T cells can eliminate B cells *in vivo* for an extended period of time (73). It has been reported that the CD19-targeted CAR-T cells persistently depleted CD19+ B cells, eliminated autoantibody production and extended life spans in a mouse lupus model (74). However, as normal B cells can also be being killed by CAR-T cells targeting B-lineage antigens, this significantly impairs the body's ability to fight disease, resulting in the requirement of immunoglobulin injections to continuously maintain immunity. Therefore, it might not be practical to treat autoimmune diseases using CAR-T to clear B-lineage lymphocytes. To circumvent this problem, the chimeric autoantibody receptor (CAAR) has been constructed based on autoantigens, this is capable of binding to the BCR of autoreactive B lymphocytes and therefore has the ability to specifically eliminate autoantigen-reactive B cells. It was reported that CAAR-T cells with the extracellular domain desmoglein3 (Dsg3) were able to eliminate Dsg3-specific B cells in preclinical models of pemphigus vulgaris (PV) (75). Compared to other B-cell depletion therapies, CAAR technology only removes autoreactive B cells, avoiding a decline in immunoglobulins and the opportunistic infections caused by B-cell clearance, and might provide an effective and universal strategy for the specific targeting of autoreactive B cells in antibody-mediated autoimmune disease (Figures 3B,C). However, there are multiple autoantigens that are attacked by the immune system and individualized differences in ADs. Therefore, not only the coverage of disease-associated antigens but also the molecular weight and spatial epitope of each antigen should be considered in the design and construction of effective CAARs to treat such diseases. Recently we observed that the depletion of PD1^+^ cells mainly removes autoreactive cells and results in a therapeutic effect in several animal models of inflammatory diseases (76), providing an encouraging approach to combat autoimmune diseases.

## Strategy 3: Rebuilding Autoimmune Tolerance Using Various Immunoregulatory Cells

Antigen-presenting cells (APCs), including dendritic cells (DCs) and monocytes/macrophages, play an important role in the regulation of innate and acquired immunity, as well as bidirectional regulation in both antigen-specific immunity and immune tolerance. Tolerogenic antigen-presenting cells (tolAPCs) comprise a specific type of APC with immunoregulatory functions (77, 78).

**Figure 4:**
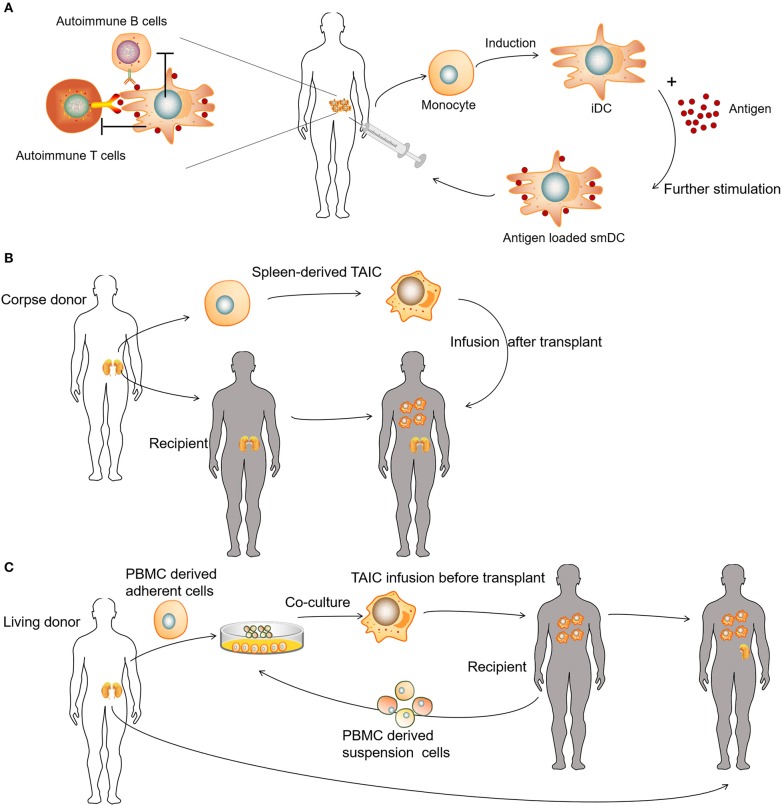
Clinical uses of various tolerogenic antigen-presenting cell (tolAPC) methods. **(A)** Patients receive autoantigen-loaded tolDCs. **(B)** Patients receive kidney transplants and corpse donor spleen-derived transplant acceptance-inducing cell (TAIC) treatment. **(C)** Patients receive living donor PBMC-derived TAICs before kidney transplants.

Tolerogenic DCs (tolDCs) are a heterogeneous pool of dendritic cells with immunosuppressive properties. In autoimmunity, DCs tend to produce pro-inflammatory cytokines and lead to the activation of autologous antigen-reactive T cells (79). The DC-induced immune response or immunotolerance is determined by the maturation state of DCs, and both immature DCs (iDCs) and semi-mature DCs have been shown to be tolerogenic (80–84). TolDCs modulate adaptive immune responses and restore tolerance through different mechanisms that involve anergy, the generation of regulatory lymphocyte populations, or the deletion of potentially harmful inflammatory T cell subsets (Figure 4A) (85–88). Dhodapkar et al. (80) first reported the ability of tolDCs to induce antigen-specific immune tolerance in healthy volunteers, using keyhole limpet hemocyanin (KLH) and influenza matrix peptide (MP) in combination with iDC injection, which led to the specific inhibition of MP-specific CD8^+^T cell effector function and the appearance of MP-specific interleukin 10-producing cells. Giannoukakis et al. (81) reported the safety of tolDCs in adult type 1 diabetic patients. C-peptide levels in some of the subjects became detectable, whereas prior to and at the time of enrollment, they were undetectable. Amezaga et al. (82) reported the treatment of refractory Crohn's disease using tolDCs characterized by high levels of IL-10 production in response to lipopolysaccharide and gram-negative bacteria, wherein three of the nine patients exhibited improved outcomes, with an increase in circulating regulatory T cells (Tregs) and a decrease in IFN-γ. Benham et al. (83) used four different citrulline peptide-loaded NF-κB inhibitor (BAY11-7082)-treated tolDCs, “Rheumavax,” for subcutaneous injection to RA patients. One month post-treatment, patients exhibited lower DAS28 scores, decreased effector T cells, increased FOXP3^+^ Tregs, and reduced serum C reactive protein, IL-15, IL-29, CX3CL1, and CXCL11, confirming the efficacy and safety of Rheumavax for the treatment of RA. In a further demonstration, Bell et al. (84) injected autologous synovial fluid antigen-loaded tolDCs into the joint cavity to treat rheumatic and inflammatory arthritis, leading to reduced inflammation and pain. Researchers have also established various optimized protocols to maintain the stability of tolDCs and enhance their lymph node-homing capacity (Table 1). Several modulating actors, such as dexamethasone and vitamin A or vitamin D3 were proven to induce DCs with stable regulatory capacity, whereas partial maturation endows tolDCs with robust antigen presentation and lymph node-homing capacity (82, 84, 89).

**Table 1:** Tolerogenic dendritic cell (tolDC)-inducing protocols.

**Disease/health**	**DC maturity**	**tolDC generation**	**Antigens pulsed**	**Further stimulation**	**Harvest**	**Route**	**References**
Healthy adult volunteers	Immature	IL-4; GM-CSF	KLH, MP	—	Day 6 or Day 7	Subcutaneous or intradermal	(80)
Type 1 diabetic	Immature	IL-4; GM-CSF; mixture of antisense oligonucleotides targeting CD40, CD80, and CD86	No antigen	—	Day 6	Intradermal	(81)
Crohn's Disease	Semi-mature	IL-4; GM-CSF; dexamethasone; vitamin A	No antigen	IL-1β, IL-6, TNF-α, and PGE2	Day 7	Intraperitoneal	(82)
Rheumatoid arthritis	Immature	IL-4; GM-CSF; Bay11-7082	Cit-peptide	—	Day 3	Joint injection	(83)
Rheumatic and inflammatory arthritis	Semi-mature	IL-4; GM-CSF; dexamethasone; vitamin D3	SF	MPLA	Day 7	Joint injection	(84)

*KLH, keyhole limpet hemocyanin; MP, influenza matrix peptide; Cit-peptide, citrulline peptide; SF, synovial fluid; MPLA, monophosphoryl lipid A*.

Unlike autologous tolDCs, donor-derived regulatory macrophages (Mregs) have also been used to induce allogeneic tolerance during organ transplantation. Transplant acceptance-inducing cells (TAICs) are primarily considered a class of immunoregulatory macrophages (90–92). In the TAIC-I clinical trial, 12 patients received renal transplants and corpse donor spleen-derived TAIC treatment (Figure 4B). An immunosuppressive regimen (including tacrolimus and sirolimus triple) was used, following TAIC treatment 5 days post-transplantation. In the majority of patients (10 of 12), the amount of supporting immunosuppressant was gradually reduced, whereas eight patients discontinued steroids at 8 weeks, and six of eight patients discontinued sirolimus at 12 weeks; only low-dose tacrolimus maintenance therapy was required subsequently (91). In the TAIC-II clinical trial, the investigators co-cultured adherent cells derived from living donor peripheral blood mononuclear cells (PBMCs) with suspension cells from the recipient PBMCs to obtain TAICs for renal transplantation (Figure 4C). Clinically, it was possible to treat four of five with tacrolimus monotherapy rather than conventional immunosuppressive regimens, whereas three of five patients were able to tolerate low-dose tacrolimus monotherapy. Although the trial failed to demonstrate the effectiveness of TAICs, treatment was able to eliminate several allogeneic reactions (92). For example, post-TAIC transplantation, the anti-donor HLA-specific antibody disappeared and, in some cases, serum immunoglobulin returned to normal levels, indicating that TAIC treatment could eliminate donor-HLA-specific antibodies (90).

In another clinical trial (93), the investigators adjusted the TAIC-II clinical trial to trace the presence of Mregs in the body. Mregs were initially trapped in the lung, and thereafter, cells were detected in circulation after 2.5 h and began to accumulate in the liver and spleen. After 22 h of Mreg administration, the signal was markedly reduced in the lung and increased in the liver, spleen, and hematopoietic active bone marrow. No signal was detected from the patient's urinary tract throughout the examination period, indicating that most infused Mregs remained alive. There is a risk of pulmonary embolism in cell therapies, and the risk is theoretically related to the number and size of the cells. However, human Mregs are larger in volume than other cells used in cell therapy, having a diameter of ~15–30 μm. As the average diameter of pulmonary capillaries is 7.5 ± 2.3 μm, it is important to consider the risk of pulmonary embolism associated with Mreg treatments (94).

Tregs are important regulatory cells that maintain peripheral immune tolerance (95–98). Claudio et al. (99) first used cultured cord blood polyclonal Tregs to treat acute GVHD (aGVHD), and 23 patients treated with Tregs exhibited a lower incidence of grade II-IV aGVHD compared to that in 108 historical controls (43 vs. 61%, *P* = 0.05); this was associated with no toxicity after infusion and no detrimental effects of infection or instances of relapse or early death. Mauro et al. (100) evaluated the effects of GVHD prevention and immune reconstitution through the early infusion of freshly isolated Tregs for 28 high-risk hematologic malignancy patients treated with HLA-haploidentical HSCT. Their findings indicated that the adoptive transfer of Tregs prevented GVHD in the absence of any post-transplantation immunosuppression, promoted lymphoid reconstitution, improved immunity to opportunistic pathogens, and did not diminish the graft-versus-leukemia effect. Natalia et al. (101) treated 12 children with type 1 diabetes (T1D) with autologous expanded Tregs. After transfusion, the number of Tregs in peripheral blood increased, and no serious side effects were observed. Most patients exhibited increased C-peptide levels and lower demand for insulin, whereas two patients were completely insulin-independent at 1 year. In another study, similar results were observed in adult subjects (102). However, a phase II clinical trial (NCT02691247) showed that autologous *ex vivo* polyclonally expanded Treg infusion failed to preserve C-peptide production 1 year after the start of treatment in 113 newly diagnosed adolescents with T1D (103). One major problem is that transferring a large number of Tregs of broad undefined specificity can potentially suppress protective immunity against tumors and infectious diseases (104). Evidence from animal models of Treg therapy has clearly shown that antigen-specific Tregs are vastly superior to polyclonal Tregs, meaning that fewer cells are needed for the desired therapeutic effect (105).

**Figure 5:**
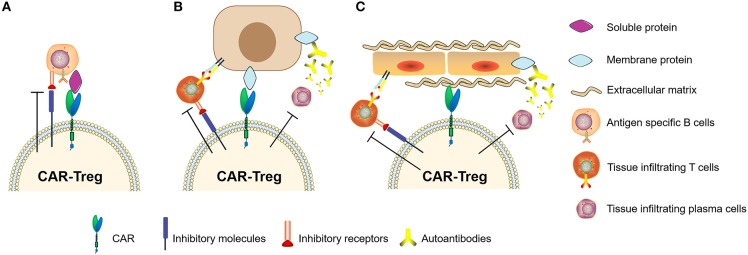
Chimeric antigen receptor regulatory T cells (CAR-Tregs) can target different types of antigens. **(A)** CAR-Tregs targeting soluble proteins, **(B)** cell membrane proteins, and inhibiting autoimmune B cells or/and T cells. **(C)** We hypothesized that CAR-Tregs could also target structural proteins to achieve tissue-specific inhibition.

In recent years, studies of Tregs engineered with chimeric antigen receptors (CAR-Tregs) have made great progress in optimizing anti-inflammatory and immune-tolerogenic responses (104, 106, 107). There are several examples of this as follows. (i) Elinav et al. (108) constructed a CAR-Treg specific for 2,4,6-trinitrophenol and demonstrated the CAR-Treg-specific effect pattern in different colitis mouse models. (ii) Fransson et al. (109) constructed CAR-Treg targeting myelin oligodendrocyte glycoproteins, which exerted a continual inhibitory effect for the treatment of EAE in mice. (iii) Dan et al. (110) constructed CAR-Tregs to target carcinoembryonic antigen to treat colitis in mice and found that CAR-Tregs can accumulate at the inflammation site in mice and inhibit colitis development. (iv) MacDonald et al. (111) constructed an HLA-A2-specific CAR (A2-CAR) and showed the inhibition of GVHD caused by HLA-A2^+^ T cells. (v) Boardman et al. (112) and Noyan et al. (113) also demonstrated that A2-CAR-Tregs could completely prevent allogeneic transplant rejection without using other immunosuppressant intervention in humanized mice. (iv) Yoon et al. (114) constructed CAR-Tregs targeting factor FVIII, which effectively inhibited T cell and B cell responses to FVIII. These results showed that specifically designed CAR-Tregs can induce immune tolerance not only by targeting cell surface proteins but also by targeting secretory proteins (Figure 5). In conclusion, CAR-Tregs display the hallmarks of cell therapy, including being target-specific, long-lasting, chemotaxic, and highly efficient for broad application in the treatment of ADs, graft-versus-host diseases, and transplant rejection. We believe that CAR-Tregs will be the next frontier in the development of anti-inflammatory and tolerogenic therapies.

Mesenchymal stromal cells (MSCs), widely distributed in various connective tissues, have multi-directional differentiation potential and strong immune regulation and tissue repair functions. Therefore, MSCs naturally play a role in modulating ADs, organ transplantation, and GVHD. MSC-related clinical trials include the treatment of diabetes, RA, Behcet's disease, organ transplantation, systemic lupus erythematosus, and others (115–119). Among them, it has been shown that MSC infusion promotes implantation in HSCT, reduces the incidence of chronic GVHD, and also plays an important role in the treatment of aGVHD (120–123). In a meta-analysis of over 200 patients to evaluate the efficacy of locally injected MSCs as a treatment for inflammatory bowel disease (IBD), more than half of patients displayed complete remission, and more than two-thirds of patients showed positive treatment responses, indicating that MSCs have considerable potential for IBD treatment (124). However, the majority of MSC-based trials for ADs are still in early phases I or II. Some have promising results and no reported toxicity to date, but phase III studies will be needed to confirm their efficacy (125, 126).

MSC sources currently used for clinical trials include bone marrow, perinatal tissue, dental pulp, and adipose tissue (127–132). Differences associated with tissue source, such as donor-related variability, cell culture system, passage number, and reagent formulation, might be important factors for the biological characteristics of MSC. These characteristics mainly include the homing ability of MSCs, viability *in vivo*, and immunosuppressive ability, which exert important influences on the therapeutic effect of tolerability. Intravenous injection is one of the most commonly used infusion methods for MSCs, which can play a systemic regulatory role. Whether MSCs can home to the site of inflammation is an important factor influencing their therapeutic effect, as these cells require interactions between tissue-specific chemokines and the corresponding receptors. In a mouse model of contact hypersensitivity (CHS), CXCR5-overexpressing MSCs show significantly increased migration ability toward CXCL13, which is highly upregulated in inflamed ears of CHS mice (133). Under certain pathological conditions, ischemic or inflammatory environments might be detrimental to the survival of MSCs. Researchers have used some proinflammatory cytokine pretreatments, such as IFN-γ, TNF-α, and IL-17, among others, to promote MSC expression of IDO and NO, which increases their immunosuppressive abilities (18, 134–138).

## Conclusion and Perspectives

Most immunoregulatory cells exhibit issues regarding survivability, stability, plasticity, and homing capacity. Cells for tolerogenic treatment can be derived from an autologous or allogeneic source. Further, the fact that autologous cells might contain gene-related defects, resulting in less effective tolerogenic therapy, cannot be ignored. Whereas, the short survival period of allogeneic cells, because of rejection, might lead to poor treatment efficacy, the prolonged persistence of allogeneic cells will raise safety concerns. After infusion, the avoidance of the transformation of immunoregulatory cells into effector cells is essential for the long-term efficacy of tolerogenic therapy. In particular, if the regulatory cells used for targeted cell therapy (e.g., CAR-Treg) are unstable and transform into inflammatory cells, they might exert an adverse effect on the disease. For treatment with Tregs, the memory Tregs induce a stronger inflammatory response than naïve Tregs (139). Therefore, not only the choice between autologous or allogeneic cells, as well as the cell type, needs to be considered, but also the stable subpopulation of cells for treatment should be considered.

Genetically modified immune cell therapy technology, such as CAR-Treg, could present a breakthrough for the treatment of ADs. Genetic modification can compensate for genetic defects to a certain extent and has advantages for therapeutic cell targeting, chemotaxis, and enhancement. Similarly, the safety of gene therapy should be carefully considered, including genetically modifying vectors, the recombination of internal and exogenous genes, the controllability of gene expression, and even ethical issues.

**Figure 6:**
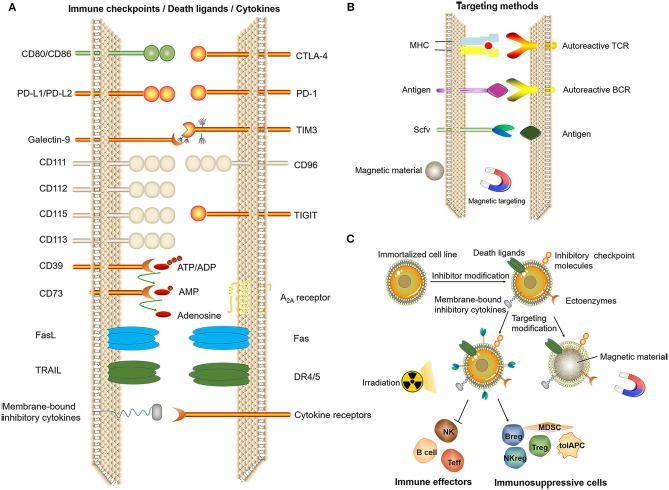
Related technologies for tolerogenic cell therapy that can be mass-produced. **(A)** Induction of immune tolerance by immune checkpoints, death ligands, (artificial) membrane-type cytokines. **(B)** Targeting methods. **(C)** Genetic modification of immortalized cell lines and screening to obtain cells with the ability to induce immune tolerance after irradiation for treatment or by using the cell membrane to bind magnetic materials, thus exerting an immunomodulatory effect at a specific site under the action of a magnetic field.

In addition, we propose two directions: immortalized cell lines and cell-like materials. Immortalized NK92 cells have been used to treat malignant tumors in several clinical trials (140). Immortalized cell lines can be mass-produced, and the product quality is easier to control. To date, there have been no studies using immortal cells for immune tolerance therapy. With the development of genetic modification technology, the immortalized cell line can be genetically engineered to express a certain level of death ligands/inhibitory checkpoint molecules/membrane-bound inhibitory cytokines or ectoenzymes to exert potent immunosuppressive effects under targeted conditions. Gamma-ray treatment of immortalized cells can induce a loss of proliferative ability while maintaining biological activity for a short period of time. Functions of regulatory cells such as Tregs do not depend on self-proliferation, and apoptotic Tregs display stronger immunosuppressive effects (141). The function of inflammatory cells might be inhibited and immunosuppressive cell functions can be enhanced by contact with artificial immortalized cells, thereby achieving the induction of immune tolerance. Therefore, immortalized cells might serve as an alternative choice for tolerogenic cell therapy (Figure 6).

As the mechanism through which various inhibitory cytokines or ligands exert immunosuppressive effects are being gradually discovered, one effective strategy might be the construction of cell-like particles based on the immune checkpoints of these known mechanisms. Some researchers have studied tolerogenic therapies using nanomaterials (142–144). Cell-like materials with individualized and regulated functions could be synthesized through biochemical methods, which can induce the apoptosis of autoreactive immune cells, thereby achieving the recovery of immune tolerance.

In conclusion, there are still many problems to be solved regarding current tolerogenic cell therapy technology. However, accumulating evidence indicates that cell-based tolerogenic treatment will undoubtedly play an important role in future medical technology.

## Author Contributions

ZW and XL wrote the manuscript. SZ, JZ, FC, and JB reviewed and edited the manuscript. All authors read and approved the manuscript.

## Conflict of Interest

The authors declare that the research was conducted in the absence of any commercial or financial relationships that could be construed as a potential conflict of interest.
